# Patient accounts of diagnostic testing for familial hypercholesterolaemia: comparing responses to genetic and non-genetic testing methods

**DOI:** 10.1186/1471-2350-13-87

**Published:** 2012-09-21

**Authors:** Gareth J Hollands, David Armstrong, Angela Macfarlane, Martin A Crook, Theresa M Marteau

**Affiliations:** 1 Health Psychology Section, Department of Psychology (at Guy’s), King’s College London, 5th Floor Bermondsey Wing, Guy’s Campus, London, SE1 9RT, UK; 2 Department of Primary Care and Public Health Sciences, King's College London, 5th Floor Capital House, 42 Weston Street, London, SE1 3QD, UK; 3 Department of Clinical Biochemistry and Metabolic Medicine, University Hospital Lewisham, London, SE13 6LH, UK

**Keywords:** Behaviour change, Genetics, Genetic testing, Health behaviour, Risk, Qualitative

## Abstract

**Background:**

Continuing developments in genetic testing technology together with research revealing gene-disease associations have brought closer the potential for genetic screening of populations. A major concern, as with any screening programme, is the response of the patient to the findings of screening, whether the outcome is positive or negative. Such concern is heightened for genetic testing, which it is feared may elicit stronger reactions than non-genetic testing.

**Methods:**

This paper draws on thematic analysis of 113 semi-structured interviews with 39 patients being tested for familial hypercholesterolaemia (FH), an inherited predisposition to early-onset heart disease. It examines the impact of disease risk assessments based on both genetic and non-genetic information, or solely non-genetic information.

**Results:**

The impact of diagnostic testing did not seem to vary according to whether or not genetic information was used. More generally, being given a positive or negative diagnosis of FH had minimal discernible impact on people's lives as they maintained the continuity of their beliefs and behaviour.

**Conclusions:**

The results suggest that concerns about the use of genetic testing in this context are unfounded, a conclusion that echoes findings from studies in this and other health contexts.

## Background

New genetic testing technologies and reported gene-disease associations have resulted in the appearance of many genetic testing services, particularly from the private sector (e.g. 23andMe [1]; Navigenics [2]). Whilst there has been criticism of the value of some of the latter [3], high expectations about the beneficial effects of providing such information are also in evidence [4].

Some commentators have suggested that in healthcare settings genetic risk factors are often perceived as carrying more weight for practitioners and patients than equivalent environmental factors [5]. Such a position is in accordance with a historic biomedical discourse in which patients with a genetic mutation develop a given illness, informed by examples such as Huntington’s disease and cystic fibrosis. Previous studies of genetic disease risk assessment, however, report mixed evidence regarding its impact. Whilst a systematic review of the psychological consequences of predictive genetic testing for Huntington’s disease and breast cancer found no evidence of adverse experiences [6], the significance of the penetrance of a genetic variant, i.e. the extent to which the genotype determines the phenotype disease state, has been highlighted in other studies. As such, genetic tests with high predictive value are regarded as different and may have more potent effects [7,8]. It has also been suggested that patients typically feel more involved with their condition when it is presented as having a genetic basis [9]. Furthermore, it has been found that the provenance of risk information influences the responses that are adjudged to be most effective, with provision of genetic risk information to individuals being associated with higher perceived effectiveness of biologically-based responses, such as taking medication, versus behavioural responses, such as altering levels of physical activity or diet [10,11].

Most of the evidence for the impact of genetic testing has involved studies of those with ‘positive’ results; there has been some, though less, research on those receiving a negative result. Yet these studies have not always been able to disentangle the impact of the genetic test itself from the diagnosis. For example, for many years the diagnosis of familial hypercholerosterolaemia (FH) has been made from the clinical picture of the disease rather than from a specific genetic test. As more recently genetic testing has been introduced in some centres, it is now possible to study the impact of the method of testing on patients’ response to such a diagnosis.

### Condition and clinical context

Familial hypercholesterolaemia is, as its name implies, an inherited genetic condition that results in high blood cholesterol and a recognised risk factor for heart disease. The importance of diagnosis is emphasised by the estimate that heterozygous FH leads to a greater than 50% risk of coronary heart disease in men by the age of 50 years and at least 30% in women by the age of 60 years [12]. No single mutation is regarded as sufficiently frequent to justify population screening except for individuals whose families have been identified as carrying the mutation. Diagnosis has traditionally been based on phenotypic factors, namely, family history of raised cholesterol and early coronary heart disease, and on clinical symptoms, including tendon xanthoma [13]. Patients are rarely seen in dedicated genetic clinics and instead are usually seen in lipid clinics that manage patients with abnormal blood lipids, irrespective of origin. Latterly, however, some clinics have begun to introduce genetic testing. This approach to testing has advantages in terms of increased accuracy and the potential for early diagnosis, but also disadvantages, such as cost and the potential impact on patients [14].

Qualitative studies that have focused on FH suggest a positive diagnosis for FH does not evoke substantive effects in patients. Senior and colleagues [15] found that those receiving genetic risk information were likely to see their results as routine and holding no greater significance than other risk factors. This is consistent with the suggestion that existing lay models readily accommodate genetic information as individuals already expect a familial component to cardiovascular disease [16]. Weiner and Durrington [17] found that individuals screened for FH do not tend to view their genes or heredity as having a major deterministic role in heart disease, or see an FH diagnosis as problematic in the long term. Existing evidence therefore suggests that there may be no systematic impact of the diagnosis within the context of FH risk assessment.

Yet receiving a genetic diagnosis and undergoing a genetic test are two different experiences and this study sought to disentangle their respective impacts by examining groups of patients with different experiences of each.

## Methods

While a qualitative approach was adopted for data collection and analysis, sampling was based on a factorial design which recruited groups of patients based on their experiences of genetic or non-genetic testing and negative or positive test results.

**Figure 1:**
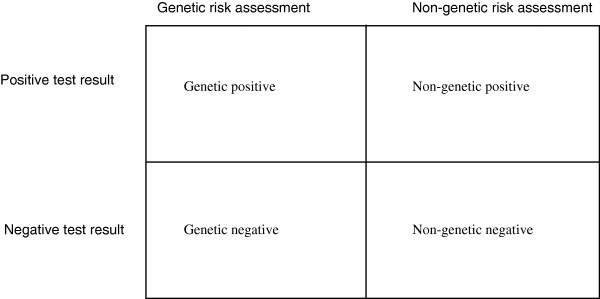
Participant sampling categorisation by diagnosis.

Clinic nurses invited all patients who were first or second degree relatives of probands with familial hypercholesterolaemia (FH) and who attended a specialist lipid clinic in one of 11 participating hospitals in the UK for diagnostic testing of FH to be contacted by the study team. Those who agreed were informed about the nature of the study and written consent was obtained. The sampling strategy aimed to recruit 40 patients in total, with 10 from each diagnostic category or cell (see Figure 1). This dictated that patients were consented and underwent baseline interviews before testing and were then followed up to assess their reaction to both the form of the test and the result. Follow-up was therefore selective to ensure the required numbers (10 patients) in each of the factorial cells. In the event, ten patients were recruited from the non-genetic negative diagnosis category, with nine, nine and eleven recruited from the other categories (respectively non-genetic positive, genetic negative and genetic positive), making 39 patients in total.

Characteristics of study participants are presented in Table 1. We did not systematically gather detailed information on participants’ unique clinical histories and pathways leading to testing at the clinic, but patients were usually identified in primary care or in cardiology services through assessment of symptoms and family history, typically involving a cholesterol test. They were then referred to specialist services for further assessment and diagnosis. As a result of the prior investigations, many patients were likely to expect a definitive FH diagnosis from the specialist service at the time of their referral. Participants in this study were therefore asked whether they had received cholesterol tests in the past and what FH test result they expected. It is apparent from Table 1 that the majority of participants in all categories had received a cholesterol test previously, consistent with the fact that participants typically accurately predicted their diagnosis.

**Table 1:** Participant characteristics by familial hypercholesterolaemia test result received

	**DNA diagnosis**	**DNA diagnosis**	**Non-DNA diagnosis**	**Non-DNA diagnosis**
	**positive**	**negative**	**positive**	**negative**
**Age (mean)**	30.9	37.3	40.7	43.9
**Gender**				
Male	5	5	7	5
Female	6	4	2	5
**Ethnicity**				
White British	9	5	5	7
Asian British	1	2	0	0
White Other	1	0	2	0
Jewish	0	1	0	0
Black-Caribbean	0	0	2	3
Greek-Cypriot	0	1	0	0
**Prior cholesterol test**				
Yes	6	6	9	7
No	3	3	0	3
**Expected test result**				
Negative	0	4	0	4
Positive	9	0	6	0
Uncertain	2	5	3	6

Consenting patients were contacted for three telephone interviews each: following the initial consultation but before receiving the results of diagnostic testing; immediately after receipt of the test results; and six months later. In total, 113 separate interviews were conducted and used for the analysis, with four intended follow-up interviews unable to be completed due to the patients being lost to contact.

All interviews were transcribed by an external transcription service and analysed using thematic analysis managed with NVivo computer software [18]. Transcripts were analysed separately by two members of the research team, with a third member of the team acting to oversee the integration of findings when analyses were subsequently compared and discussed. There was a high level of comparability between the emergent themes that were identified. The determination of themes drew broadly from all sampling categories. Respondents are identified in the text as follows: undergoing risk assessment that included genetic testing (DNA) or risk assessment not including genetic testing (NonDNA); diagnosis (positive or negative); participant number by site (01–11); timepoint of interview (1, 2 or 3).

The study had ethical approval from Huntingdon Research Ethics Committee (06/Q0104/16).

## Results

Five principal themes emerged in the analysis. These are described below.

### Continuity and normalisation

The main feature of patients’ responses to either a positive or negative diagnosis across all four groups was one of normalisation and continuity, that is, the results of the tests offered no significant challenge to their identities or their lives in the future. This was reflected both in the way they regarded the test results and more generally the implications of a diagnosis.

Many patients in all four categories had realistic expectations of the results of diagnostic testing (i.e. they had an accurate sense of the likely diagnosis) whether it included a genetic test or not. None of the patients who ultimately received positive test results had expected a negative test result, prior to receiving their results, and vice versa. Having had relatives with FH or high cholesterol they knew they were at risk, had undergone cholesterol tests over preceding years, and often talked about the familial context of their risk. If these earlier cholesterol tests had revealed high values then they had assumed that, like their relatives, they had FH. Conversely, if their cholesterol values had been within the normal range, they had assumed they were unaffected. Hence, very few patients expressed much surprise at the outcome of their diagnostic assessments. This did not vary between those whose diagnostic assessments included a genetic test and those whose did not.

Prior to receiving their results, patients typically expressed confidence in anticipating the likely outcome, and indicated that they had already accepted it:

"(Interviewer: How have you been thinking about the test before coming to the hospital?) I’ve not really been too concerned about it to be honest. I mean I’ll be honest, last time I had my cholesterol checked it was 3.5, it was low. I think my brother’s got it because he tends to have a lot of the symptoms."

"(NonDNA, negative, 01, time 1)"

"(Interviewer: So how would you feel if the results that come back show that you do have FH?) Well I would know that anyway so it wouldn’t surprise me."

"(NonDNA, positive, 09, time 1)"

"I would be a bit shocked actually because I’m so convinced I don’t have it."

"(DNA, negative, 03, time 1)"

This expectation of what the test result might reveal was also reflected in later responses to the test result.

"I wasn’t surprised because I didn’t really think I had it to be honest."

"(NonDNA, negative, 01, time 2)"

"I thought they would be anyway so it just… I was pleased obviously but deep down I thought that would be the case."

"(DNA, negative, 03, time 2)"

"Beforehand I knew they would be clear anyway. I haven’t had any problems of history with these sort of problems anyway so I was fairly confident they were going to be alright."

"(DNA, negative, 04, time 2)"

"I wasn’t surprised. I know there has been a family history of it so it was a 50/50 chance of me having it or not having it."

"(DNA, positive, 01, time 2)"

"It just confirmed what I expected really. I need to get my blood tests done again for the cholesterol and just keep an eye on it."

"(DNA, positive, 02, time 2)"

A sense of acceptance of the implications of the results for people’s lives and their ongoing health was also apparent in responses both before and after receiving the test results. Some patients, however, did propose some small adjustments even though the results had been predictable.

"If my body’s producing it then I’ll just have to learn to live with it really and it’s not stopping me from doing my everyday life. I can still manage to go to work and you know look after my family etc. so it’s not something stopping me in any way. I don’t know I think I’ll just be the same really."

"(NonDNA, positive, 08, time 1)"

"I don’t think it would have a major impact, I’d just have to change my lifestyle slightly, eat a bit more healthily."

"(DNA, negative, 05, time 1)"

"It wouldn’t have that much of an impact, like change my life that much. It would worry me slightly about what I’d be eating and that sort of stuff, my diet, but I’m not really a fussy eater so."

"(DNA, positive, 03, time 1)"

"To be honest with you it wouldn’t make me feel any different to how I do now because I am almost convinced that that is the case anyway. It’s something that I’ve lived with for a long time anyway. It doesn’t really affect my health, you know day to day living."

"(DNA, positive, 04, time 1)"

This sense of simply getting on with the routine of their lives was also reflected in their responses after the test results. Again, whether the test was a genetic one or not did not seem to make any difference.

"As I say I don’t, it’s not something I’m really worried about. I mean… I felt sort of positive that I wouldn’t have it prior to the test…I’ve not really sort of dwelled on it and sort of… yeah I’ve just sort of got on with life basically."

"(NonDNA, negative, 07, time 2)"

"(Interviewer: So how did you feel about your results when you received them at the last appointment?) Okay. I’ve known that I’ve had high cholesterol so I was just pleased that they were increasing the dosage (of Pravastatin)."

"(NonDNA, positive, 09, time 2)"

"I continue to do what I did anyway, which is watch my diet and exercise regularly, and since then, obviously, medication’s come onboard, so I’m fine with it."

"(NonDNA, positive, 01, time 3)"

"I was expecting them so I wasn’t too upset. I wasn’t too shocked or alarmed. I was kind of thinking I was going to have them because of my mum and my sister having them so I was quite well prepared for it. I’m alright about it. I just accept the fact that I’ve got it and just deal with it and make it not an issue in my personal health."

"(DNA, positive, 10, time 2)"

### Reduction of uncertainty

While the diagnosis did not seem to have a great impact on these patients’ lives, they all had immediate reactions which revealed that they were managing to adjust and incorporate this new information into their biographical cognitive framework. For example, many patients reported that the diagnostic test, whatever its outcome, had reduced the uncertainty that surrounded their health status. While most reported clear expectations of what the test might reveal, the actual result removed any residual doubt. Whether a positive or negative test result, from either a genetic or non-genetic test, the removal of uncertainty was generally seen as a good thing, simply because certainty was preferable.

"It’s best for me knowing now that I haven’t got to sort of alter my lifestyle greatly to sort of try and fight something, through no fault of my own."

"(NonDNA, negative, 06, time 2)"

"(Interviewer: So what do your test results mean for your health now?) They’re peace of mind you know."

"(NonDNA, negative, 08, time 3)"

"I guess it’s a relief in a funny way because I had an answer to what was quite a surprising medical condition that I had, which is the stroke that I had…So at least I know now and can take preventative measures"

"(NonDNA, positive, 06, time 2)"

"I mean the whole experience has been a positive thing. Obviously it’s quite scary to start with because you don’t really want to know but it is better to know and to get it sorted. I am relieved."

"(DNA, negative, 02, time 2)"

### Lifestyle reinforcement

Another effect of the diagnosis was to incentivise patients to maintain healthy lifestyles. This was particularly salient for those receiving a positive diagnosis but even in those receiving a negative report there was still an increased awareness that healthy lifestyles were of general benefit. In this way, diagnosis had a reinforcing effect on health-related behaviours but generally seemed not to induce new ones (other than changes to prescribed medication).

Receipt of a negative test result led patients to reflect upon their past and future behaviour. Those undergoing a non-genetic assessment perceived that the risk of having FH was reduced by the adoption of a healthy lifestyle and the prognosis of the disease improved by a healthy lifestyle. The failure to diagnose FH was thus perceived as a vindication of their prior behaviour.

"(Interviewer: What do these test results mean for your health now?) That I carry on eating a healthy diet and making sure I get exercise. I have to continue to monitor my diet and try to keep healthy."

"(DNA, positive, 02, time 2)"

"It just means that I am obviously doing something right and carry on the way I’m going. … if I just carry on doing what I’m doing, keep exercising the way I do and eat what I eat then I should be okay."

"(NonDNA, negative, 06, time 2)"

"Well it shows that I am looking after myself."

"(NonDNA, negative, 07, time 2)"

"It just reinforces that I want to continue to have a healthy lifestyle."

"(NonDNA, negative, 10, time 3)"

Patients who had a negative diagnosis of FH through a genetic test also attributed their negative diagnoses to healthy living.

"(Interviewer: What does the result mean to you in terms of your health?) That I'm looking after myself, that I'm eating properly, looking after my cholesterol. Just means I need to continue what I’m doing, try and stay away from the fatty foods and the unhealthy stuff and maintain a healthy balanced diet."

"(DNA, negative, 04, time 2)"

"Perhaps it has something to do with the lifestyle that I've had, because up until like my 30s I was quite an active person, I was quite fit, I was in the Army so I suppose that helps. Whereas my dad he… I think the last time he did anything really physical like that he was at school."

"(DNA, negative, 06, time 2)"

"I was always interested in sports as a youngster and I’ve kept myself busy, looked after myself and so it didn’t allow any sort of fat, if you like, to develop as much."

"(DNA, negative, 06, time 3)"

Patients with negative diagnoses in both genetic and non-genetic assessment groups intended to continue to behave healthily in the future.

"Just again watch what I’m eating really, you know, continue to watch what I’m eating even though I haven’t got it hereditary, you know you can still get it by eating, you know, unhealthily and it’s basically just a healthy lifestyle. Just to continue trying to be healthy and live a healthy lifestyle and eat healthily."

"(NonDNA, negative, 04, time 2)"

"I wouldn’t say so, no but you can’t be too complacent as in oh right I’ve not got it so I can go out and be really silly and eat lots of silly things, you know you’ve still got to be realistic and think ‘well you know I still have to watch what I eat and do exercise’."

"(NonDNA, negative, 09, time 2)"

"I will do my damnedest to have more exercise, which I am very lazy now because I do tend to use the car to go everywhere even up to the shop. In the past where I couldn't drive it was quite naive, ride a bike or walk. I must admit I have been quite good in the last month or so, I do walk a lot more."

"(DNA, negative, 03, time 2)"

"Try not to eat too much junk and get more healthy."

"(DNA, negative, 05, time 2)"

### The importance of FH relative to other conditions

The way in which the diagnosis (or non-diagnosis) was incorporated into everyday lives was also illustrated by a few patients who in the course of the interview revealed that they had other illnesses which were clearly of greater salience. For example, one patient had been diagnosed with hepatitis while another had a long-standing problem of psoriasis. In both cases it was clear that these individuals viewed their other illness as more important than their risk of heart disease.

"Unfortunately, I've got other problems so, although I've got rid of that I've got other things to contend with anyway. Yeah I'm pleased with that (the FH result) but I've found out I've got Hep C so I go for injections for that so yeah."

"(DNA, negative, 03, time 3)"

However, the process of engagement with clinical services and accompanying focus on their health associated with FH testing and treatment appeared to enable patients to have greater confidence about addressing other medical concerns. The patient with psoriasis expressed this as follows.

"One thing that’s changed in my life is that I seem to be sort of at the doctors a lot. And now when you find out there is something wrong I think that I am a little more aware of my health. If something happens I do tend to think “I’ll go and see the doctor about that.” and get it sorted out. So I have psoriasis and things like that, so I try and pay more attention to that and see if I can sort it out. But it’s interesting, I feel like at the age of 40 I’ve had an extremely good medical, I mean they’ve taken blood tests for everything you can think of."

"(NonDNA, positive, 02, time 3)"

Another patient had recently had a triple heart bypass and it was clear that the FH diagnosis was just a part of the overall recovery process and not a predominant focus.

"(Interviewer: Do you talk about FH with your partner or family?) Not since the bypass and that, it’s just the road to recovery really, I haven’t really mentioned… we haven’t really discussed it."

"(DNA, positive, 09, time 2)"

Overall, the type of test did not seem to affect the saliency of other illnesses.

### Social impact

While the results of the diagnostic tests did not seem to have a discernible direct impact on patients’ self-perceptions, it did at times seem to affect their relationships with others. Once they had a positive diagnosis of FH, their social status changed in as much as their strict lifestyle regime was now based on a formal medical category rather than a general motivation to keep healthy. Some patients were therefore surprised when they revealed their FH status (or even their appointment for testing) to others to find general level of concern which was far greater than their own. These effects did not appear to differ by the method of testing.

"I’m quite a relaxed person but it was the reaction from my peers, my friends and my wife. I’m not secretive but I don’t go on about things very much like that. So when I said I had this appointment and stuff like that at dinner parties and at the pub with friends they were more shocked, they were like “Oh my God”. The fact that I was in any way not perfectly healthy was, it was shocking to them."

"(NonDNA, positive, 02, time 1)"

Several patients described difficulty explaining to others their dietary choices in social situations. This at times caused some embarrassment as they have to explain their behaviour in terms of having a medical condition rather than being fussy or needlessly watchful.

"I mean initially when you say you’re on a low fat diet people assume you’re trying to lose weight or something and they can be quite negative. I’m quite slim and they assume I’m being stupid and trying to starve myself."

"(DNA, positive, 02, time 2)"

"Some people comment on the things I eat. And then I’m like “well actually I have to eat this because I’ve got FH and I have to watch my diet”."

"(DNA, positive, 07, time 3)"

At other times, revealing the diagnosis of high cholesterol led peers to comment negatively on the behaviour of the patient.

"If you are in discussions with us and you sort of mention that you have high cholesterol, immediately everybody says well it’s due to eating rubbish, it’s due to eating fatty foods, no exercise, things like that. So people assume that because you say you’ve got high cholesterol it’s as a result of things that you’ve done."

"(NonDNA, positive, 01, time 2)."

Another patient found that having a positive diagnosis for FH allowed them to respond to similar negative comments about their health and behaviour.

"I guess the benefit of being able to say “I’ve got FH” means that you can say, “actually it’s not just that I’m a fat boy” but yeah you can actually say “well actually it’s not” because people say, “well if you ate salad and did some exercise” and you can say “actually, it’s not just that it’s…”."

"(NonDNA, positive, 03, time 2)"

## Discussion

Receipt of a formal (i.e. clinically determined and intended as definitive) diagnosis of familial hypercholesterolaemia (FH) had minimal impact on these patient's lives. The dominant response was one of continuity of both their beliefs and behaviour. Some patients did report some minor effects on their social relationships, broadly consistent with the limited prior research into the specific impact of an FH diagnosis. In addition, there was no discernible difference in impact for those whose diagnostic assessment included a genetic test compared with those whose assessment was made without genetic testing. These findings echo those from the comparison study of FH diagnosis from the perspective of health practitioners [19]. In that context, while practitioners appeared aware of and interested in the genetic element of FH, genetic testing did not play a major part in the diagnostic process.

These findings fit within psychological theories of self-regulation that describe the ways in which individuals are able to maintain emotional consistency whilst dealing with threats to their well-being [20]. Within this study, there was evidence of individuals both engaging in behaviours to reduce threats to health and also adapting cognitively to minimise the impact of the information that was presented to them. Regarding the former strategy, largely irrespective of the nature of their results, patients were found to use the results as evidence of the need to maintain healthy lifestyles, for example by engaging in physical activity and monitoring dietary intake. Regarding the latter, prior to testing patients were typically highly aware of their increased risk and of the nature of the testing process, and often had successfully predicted the outcome, allowing for relatively ready adjustment. They were commonly able to assimilate and integrate the test result, and even derive a sense of relief, again usually irrespective of the outcome of the assessment, in part due to regarding it as a means to reduce or remove ongoing uncertainty about their health.

Whilst the scope of the data collection process used in the current study was substantial, the results inevitably raise a question of the extent to which they can be generalised beyond the current context. To begin to address this, it is instructive to examine the results in the light of a series of systematic reviews which have assessed the impact of genetic test results on cognitions, emotions and behaviour. These reviews display considerable consistency in suggesting that the receipt of such results typically has little discernible impact. For example, a Cochrane Review that examined behavioural effects of DNA-based disease risk assessment [21] found little evidence of any effect on risk-reducing health behaviours, running contrary to the commonly-voiced belief that such risk assessments motivate behavioural changes. A systematic review of the emotional impact of screening (including genetic screening) for disease risk [22] found no evidence of enduring adverse effects on depression, anxiety, general distress or quality of life. Finally, regarding impact on patients’ cognitions, Collins and colleagues [23] found no effects of personalised genetic risk information on perceived control over the risk in either the short or longer term, with fatalistic responses not seemingly being engendered. In sum, in spite of widespread beliefs amongst healthcare providers that genetic screening is likely to have systematic and potent emotional and behavioural effects [24-26] the evidence increasingly suggests that this is unlikely to be the case.

Instead of a generalisable pattern irrespective of the degree of conferred risk, impact could be a function of the size of the conferred risk. Much of the evidence to date, including that derived from the previously described systematic reviews, has focused primarily on common complex diseases (e.g. cancers, diabetes, heart disease). For most such conditions, the penetrance of implicated gene variants is relatively low [27], but if penetrance is high and thus the presence of a specific gene variant indicates a high probability of disease, then a greater impact may be expected. Importantly, however, the current study did not find evidence for this in the context of FH, which is linked to genetic variants of high penetrance, conferring cumulative risks of coronary heart disease of more than 50% by age 50 in men and at least 30% by age 60 in women [28,29]. An additional factor to consider is the severity of health consequences associated with any genetic result. Again, there is no evidence of such effects. For example, an aforementioned review included five studies of the emotional consequences of predictive genetic testing for Huntington’s disease, which has very serious health consequences and is not regarded as treatable (unlike FH) [6] found no evidence of adverse emotional consequences in individuals found to be carriers.

## Conclusions

Giving first and second degree relatives a formal FH diagnosis had minimal impact on their lives. Using an innovative design, it was also possible to show that there was no discernible difference between those for whom genetic tests formed part of the diagnostic procedures compared with those for whom they did not. This suggests that concerns about the use of genetic testing in this context are unfounded, a conclusion that echoes findings from studies in this and other health contexts.

Research highlights are listed in Additional file 1.

## Competing interests

All authors declare that they have no competing interests.

## Authors’ contributions

DA and TMM are the Principal Investigators for the study. AM and MAC recruited study participants. AM interviewed participants. GJH, DA and TMM were responsible for the analysis. All authors drafted the manuscript and read and approved the final version.

## Research governance

Ethical approval for the study was obtained (REC: Hertfordshire 1: 06/Q0201/19). R&D approval was obtained from all hospitals through which the participants were contacted and from the South London and Maudsley Trust, which covers the academic department of psychology at the Institute of Psychiatry, Kings College London, the employer for the researchers.

## Pre-publication history

The pre-publication history for this paper can be accessed here:

http://www.biomedcentral.com/1471-2350/13/87/prepub

## Supplementary Material

Additional file 1 Research highlights. Click here for file
