# Supplementary material for: Patient accounts of diagnostic testing for familial hypercholesterolaemia: comparing responses to genetic and non-genetic testing methods
Source: BMC Med Genet. 2012 Sep 21;13:87. doi: 10.1186/1471-2350-13-87 (PMC3495051; doi:10.1186/1471-2350-13-87)
Supplement: Additional file 1 — Research highlights. [file 1471-2350-13-87-S1.doc]

**Patient accounts of diagnostic testing for familial hypercholesterolaemia: comparing responses to genetic and non-genetic testing methods**

**Research highlights**

- This paper looks at patients’ experiences of diagnostic testing for familial hypercholesterolaemia.
- With the increasing use of genetic testing methods in this context, it is now possible to examine their impact.
- Being given a formal diagnosis was found to have minimal discernible impact, whether or not genetic information was used.
- This suggests concerns about the use of genetic testing in this context are unfounded, echoing findings elsewhere.
